# The role of myristoylation in the membrane association of the Lassa virus matrix protein Z

**DOI:** 10.1186/1743-422X-3-93

**Published:** 2006-11-05

**Authors:** Thomas Strecker, Anna Maisa, Stephane Daffis, Robert Eichler, Oliver Lenz, Wolfgang Garten

**Affiliations:** 1 Institut für Virologie der Philipps-Universität Marburg, Hans-Meerwein-Str. 3, 35037 Marburg, Germany; 2 Washington University School of Medicine, Department of Infectious Diseases, Box 8051, 660 S. Euclid Avenue, St Louis MO 63110, USA; 3 Abbott GmbH & Co KG, Max-Planck-Ring 2, 65205 Wiesbaden, Germany; 4 Tibotec BVBA, Gen De Wittelaan L 11B 3, 2800 Mechelen, Belgium

## Abstract

The Z protein is the matrix protein of arenaviruses and has been identified as the main driving force for budding. Both LCMV and Lassa virus Z proteins bud from cells in the absence of other viral proteins as enveloped virus-like particles. Z accumulates near the inner surface of the plasma membrane where budding takes place. Furthermore, biochemical data have shown that Z is strongly membrane associated. The primary sequence of Z lacks a typical transmembrane domain and until now it is not understood by which mechanism Z is able to interact with cellular membranes. In this report, we analyzed the role of N-terminal myristoylation for the membrane binding of Lassa virus Z. We show that disruption of the N-terminal myristoylation signal by substituting the N-terminal glycine with alanine (Z-G2A mutant) resulted in a significant reduction of Z protein association with cellular membranes. Furthermore, removal of the myristoylation site resulted in a relocalization of Z from a punctuate distribution to a more diffuse cellular distribution pattern. Finally, treatment of Lassa virus-infected cells with various myristoylation inhibitors drastically reduced efficient Lassa virus replication. Our data indicate that myristoylation of Z is critical for its binding ability to lipid membranes and thus, for effective virus budding.

## Background

Lassa virus (LASV) belongs of the family of *Arenaviridae*. Based on phylogenetical, serological, and geographical findings, this family can be subdivided into two groups: The Old World group includes the prototype of this family, the lymphocytic choriomeningitis virus (LCMV) as well as Lassa virus. The New World group (also known as Tacaribe complex) includes other important human pathogens like Machupo virus, Junin virus, Guanarito virus and Sabia virus which are responsible for hemorrhagic fever outbreaks in South American countries. LASV is the causative agent of human Lassa fever, a viral hemorrhagic fever disease that is endemic in certain countries of West Africa. As a potential bioterrorism threat and due to the lack of an effective, safe therapy, Lassa virus has emerged as a worldwide concern.

Lassa virus is an enveloped virus that contains a bi-segmented single-stranded RNA genome. Each RNA segment encodes two viral genes in an ambisense coding strategy separated by an intergenic region. The small RNA (S-RNA) segment encodes the nucleoprotein NP and the glycoprotein precursor preGP-C which undergoes co- and post-translational cleavage events in order to obtain its mature form [1-5]. The large RNA (L-RNA) segment encodes the RNA-dependent RNA polymerase L and the Z protein [6,7].

**Figure 1:**
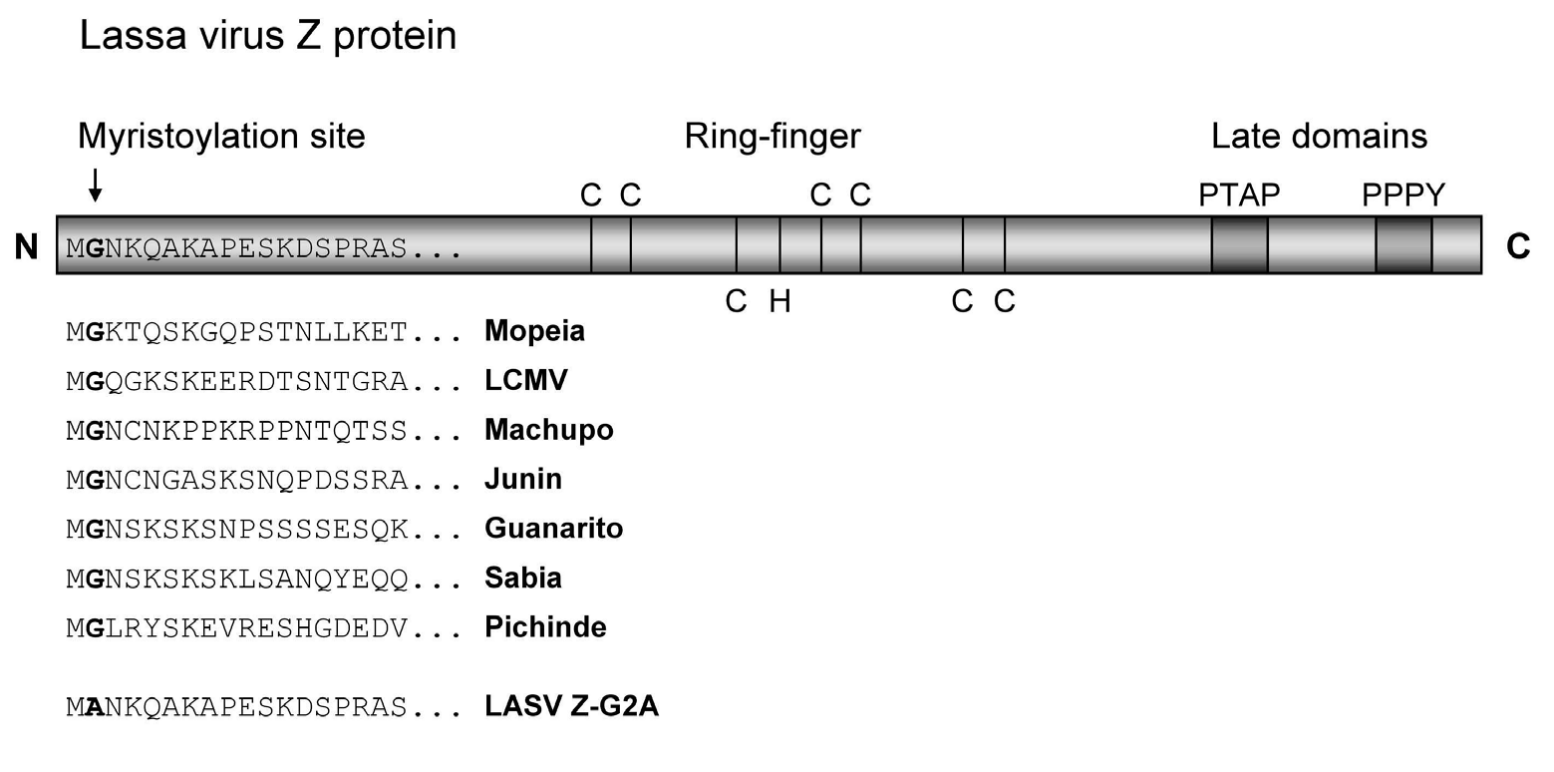
**Schematic representation of LASV Z protein**. Amino acids associated with particular structures and functions are shown in single letters. The myristoylated glycine residue at amino acid position 2 is shown in bold letters. Cysteine- and histidine residues forming the RING finger domain (C, cysteine; H, histidine) as well as the late domain motifs PPPY and PTAP (P, proline; Y, tyrosine; T, threonine; A, alanine) are indicated. Alignment of the N-termini of Old and New World arenavirus Z protein sequences are shown. Conserved glycine residues at position 2 are highlighted in bold letters. To generate the non-myristoylated mutant Z-G2A of LASV Z protein, site-directed mutagenesis was used to change the N-terminal glycine codon to an alanine codon.

Although the information available so far indicates multiple functions of Z, the role of Z during different stages in the arenavirus life cycle is still poorly understood. Several cellular factors have been described to interact with Z, e.g. the promyelocytic leukemia protein (PML), the nuclear fraction of the ribosomal protein P0, and the eukaryotic translation initiation factor 4E. However, the biological role of these interactions still remains largely unknown [8-10]. Z also exhibits a dose-dependent inhibitory effect on RNA synthesis, and cells expressing the Z protein are resistant to LCMV and Lassa virus infection [11,12]. In addition to its multifunctional regulatory role in the arenavirus life cycle, Z is also a structural component of the virus particle [13]. It has been shown that Z is the arenavirus counterpart to matrix proteins found in other negative-stranded RNA viruses [14,15]. In Lassa virus-infected cells, Z is mainly localized at the inner surface of the plasma membrane. Z protein of Lassa virus interacts with lipid membranes and can form enveloped particles that are similar in diameter and morphology to those particles released from Lassa virus-infected cells [15,16]. Furthermore, using biochemical methods we demonstrated that Z is highly membrane-associated. Membrane-detachment studies of Z revealed that treatment with chaotropic agents which characteristically remove most peripheral membrane proteins, had no effect on the association of Z with membranes [15]. The nature of this tight membrane interaction is still unknown since Z is lacking a characteristic hydrophobic transmembrane domain. Alignment of the amino acid sequences of several Z proteins revealed the presence of a putative myristoylation site at the glycine position 2 in context of a potential myristoylation signal motif that is highly conserved among New and Old World arenavirus Z proteins (Fig. 1). It has been shown recently that both LCMV and LASV Z are targets for attachment of myristic acid [17]. Mutation of the glycine at position 2, thereby disrupting the myristoylation signal prevented the incorporation of myristic acid. As a consequence Z-mediated budding activity was impaired [17]. Further evidence of the importance of myristoylation in arenavirus budding was provided by studies of Cordo *et al*. [18] showing that treatment with myristic acid analogs inhibited the replication of the New World arenaviruses Junin and Tacaribe. Thus, myristoylation of arenavirus proteins seems to be a crucial modification necessary for efficient virus replication.

N-myristoylation is a co-translational event in which myristate (a 14-carbon fatty acid) is covalently attached to an N-terminal glycine. This reaction is catalyzed by the enzyme N-myristoyl transferase (NMT) using myristoyl-CoA as a substrate upon removal of the initiator methionine by a methionine aminopeptidase [19]. N-myristoylation is a common lipid modification of proteins in eukaryotes and essential for the function of proteins involved in many cellular pathways of signal transduction, apoptosis and oncogenesis [20-24]. In addition, an increasing number of viral and bacterial proteins have been also reported to be myristoylated [25-27]. Modification of viral proteins by myristic acid plays an important role at different stages of the virus life cycle [28-36]. N-myristoylation can influence protein-protein interaction and the conformational stability of proteins as well as mediating membrane targeting and binding [22].

In this study, we investigated the contribution of N-myristoylation to the membrane binding ability of the Lassa virus Z protein. We demonstrate that myristoylation is required for efficient binding of Z to cellular membranes. Using a flotation assay we show that the mutant Z-G2A in which the amino-terminal glycine residue was changed to alanine exhibited a strongly reduced membrane binding affinity compared to wild-type Z protein. As a consequence, the budding activity of this mutant was impaired. Interestingly, the removal of myristic acid also affected the cellular localization of Z indicating that myristoylation is important for Z targeting to specific cellular regions. Finally, treatment of LASV-infected cells with myristoylation inhibitors resulted in significant decrease of LASV particle release. Our data indicate that myristoylation of Z is critical for its binding ability to lipid membranes and thus, for effective virus budding.

## Results

### Myristoylation of Z is not required for stability

**Figure 2:**
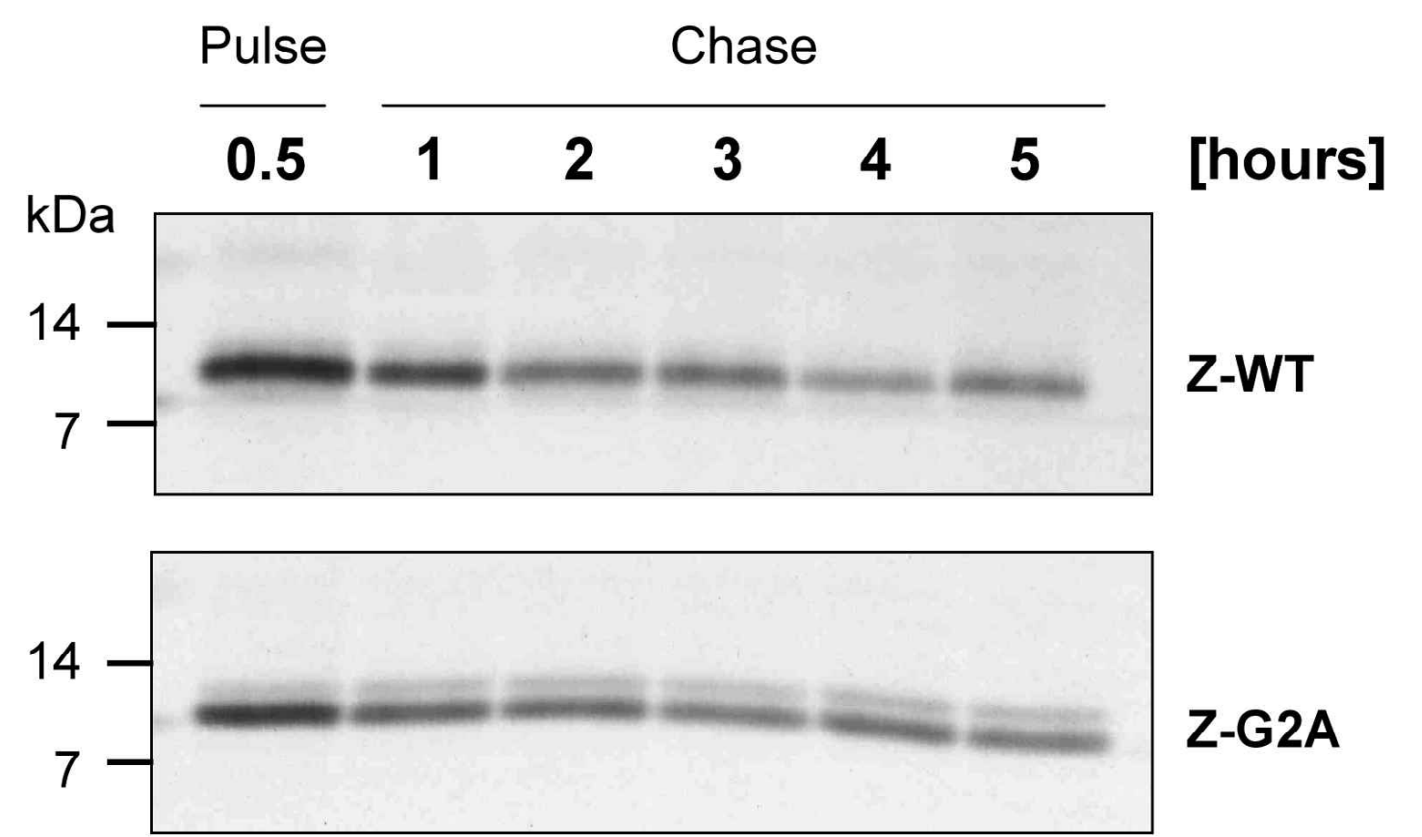
**Stability of LASV Z in the presence or absence of myristic acid**. Huh7 cells were transfected with the pCAGGS vector encoding Z-WT and Z-G2A mutant, respectively. Cells were metabolically labelled with ^35^S-methionine/-cysteine for 30 min and chased for various time intervals as indicated. Samples were precipitated using anti-Z-antiserum, subjected to SDS-PAGE on 15% polyacrylamide gels and analyzed using a FUJI BAS 1000 BioImaging analyser system (raytest).

In addition to its role in membrane anchoring, myristoylation was also reported to contribute to the stability of proteins [37]. To compare the stability of wild-type Z and a non-myristoylated mutant, we constructed a mutant, designated Z-G2A, in which the glycine at position 2 was exchanged to alanine (Fig. 1). This mutation disrupts the myristic acid attachment site and does not allow incorporation of myristic acid [17]. To address whether the removal of myristic acid affects the stability of Z and therefore undergoes faster degradation, we performed pulse-chase experiments of Z-G2A and Z-WT for up to 5 h. As shown in Fig. 2, both Z-WT and Z-G2A were expressed to similar levels and no significant change in the stability was observed. Therefore, we conclude that the removal of the myristic acid has no effect on the overall stability of Z and, thus, the myristoyl group in Z is not involved in intramolecular stabilization. Interestingly, a second band was observed in the Z-G2A construct which appeared with a molecular mass slightly higher than the main band. However, the identity of this band remains to be determined.

### Myristoylation mediates efficient membrane association of Z

**Figure 3:**
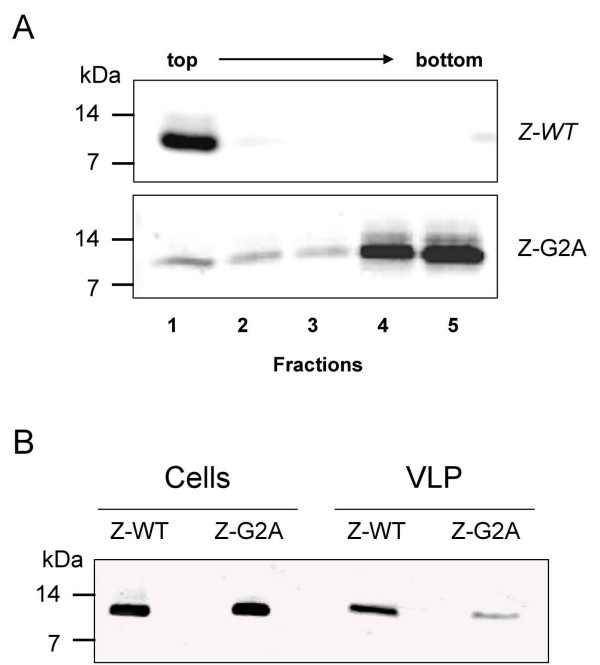
**A. Membrane association of LASV Z wild-type and Z-G2A mutant**. Huh7 cells were transfected with wild-type Z and Z-G2A mutant, respectively and 24 h post-transfection, flotation analysis was performed as described in *Materials and Methods*. Aliquots of the fractionated gradient were subjected to SDS-PAGE followed by immunoblotting using specific antisera against Z. Visualization was carried out with Odyssey™ Infrared Imaging System using a goat anti-rabbit secondary antibody labelled with Alexa680. **B. VLP formation of Z-WT and Z-G2A**. Cellular supernatants of Z-WT- or Z-G2A-transfected Huh7 cells were collected after 48 h, freed from cellular debris and then pelleted through a 20% sucrose cushion by ultracentrifugation (UC). UC-pellet containing VLPs and cell lysates as a control were dissolved in SDS-PAGE sample buffer and analyzed by SDS-PAGE and immunoblotting.

We have previously shown that solitary expressed Z has an intrinsic membrane-binding capacity [15]. Up to now the mechanism by which Z interacts with cellular membranes is still unknown. However, myristic acid attachment has been shown to play an important role in plasma membrane targeting and binding [36,38,39]. Thus, we wanted to investigate the role of myristoylation in the ability of Z to bind to lipid membranes. We performed membrane flotation analysis of the Z-G2A mutant in comparison to wild-type Z protein as described previously [15]. Huh7 cells expressing either Z-WT or Z-G2A were disrupted and the post-nuclear supernatants were subjected to flotation. Cellular membranes and membrane-associated proteins float to the OptiPrep-TNE interface during ultracentrifugation whereas soluble proteins remain at the bottom. Fractions were collected from the top of the gradient and analyzed by SDS-PAGE with subsequent Western Blotting. As described before, wild-type Z efficiently floated into the interface between 30% OptiPrep and TNE (fraction 1) showing that the Z protein is associated with cellular membranes (Fig. 3A). In contrast, the exchange of glycine to alanine at position 2 resulted in a clear shift of a major fraction of Z-G2A from a membrane to a cytosolic localization. Thus, we conclude that myristoylation contributes to efficient membrane binding of Z.

### Mutation of the glycine at position 2 is critical for LASV Z-driven VLP formation

For a number of viruses it has been shown that stable association of viral proteins with cellular membranes is required for virus assembly and production of virus particles. Lassa virus Z protein is sufficient to drive the release of Z containing VLPs that are surrounded by a lipid envelope [15]. Therefore we analyzed whether membrane binding through myristoylation is a prerequisite for Z-induced budding. To test this, supernatants of Huh7 cells expressing Z-WT or Z-G2A were subjected to ultracentrifugation (UC) through a 20% sucrose cushion. The UC-pelleted VLPs and cell lysates were analyzed by SDS-PAGE followed by immunoblotting using Z-specific antisera. As shown in Fig. 3B, the amount of Z released into the supernatant of Z-G2A expressing cells was significantly decreased compared to wild-type Z although proteins were expressed at comparable levels. Thus, consistent with the report of Perez et al. [17] mutation of the glycine at position 2 results in reduced Z-mediated formation of VLPs. Our data indicate that membrane-binding of Z is an important prerequisite to fulfill its budding function.

### Z-G2A mutant exhibits altered cellular localization

**Figure 4:**
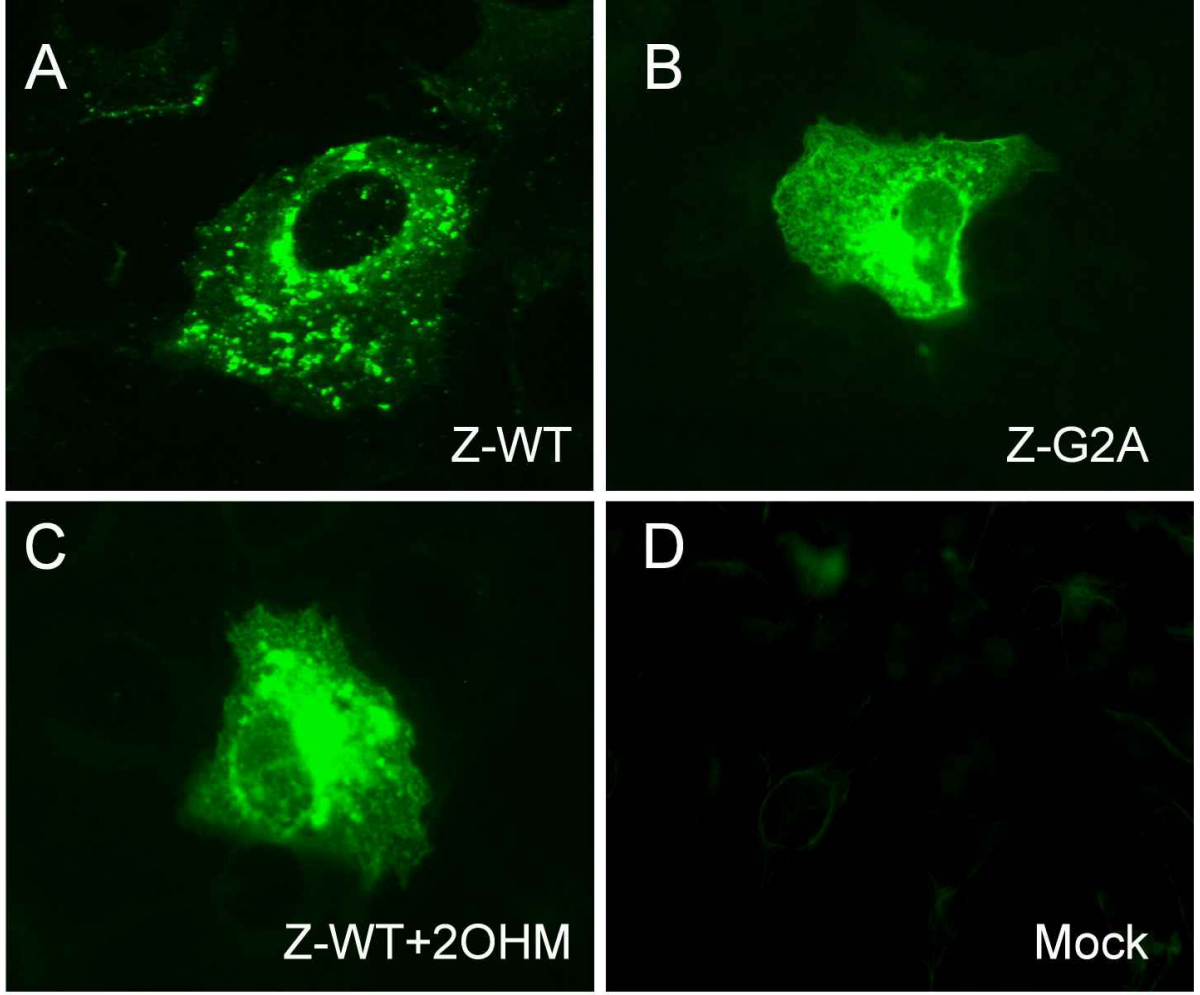
**Subcellular localization of Z-G2A and Z-WT in the presence or absence of 2 OHM**. Vero cells expressing **A**. Z-WT **B**. Z-G2A **C**. Z-WT in the presence of 2 OHM (100 μM final concentration) **D**. Mock transfection. Cells were fixed with acetone/methanol and incubated with an anti-Z polyclonal antiserum and a FITC-conjugated secondary antibody.

Next, we wanted to determine whether the loss of myristoylation affects the subcellular localization of Z. Therefore, we performed indirect immunofluorescence microscopic analysis in order to determine the cellular distribution of Z-G2A and Z-WT in the presence of the myristoylation inhibitor DL-2-hydroxymyristic acid (2 OHM), an inhibitor of N-myristoyltransferase. Consistent with our previous data, wild-type Z was distributed in a punctuate fashion throughout the cytoplasm including localization at the plasma membrane as well as the perinuclear region (Fig. 4A). In contrast, cells expressing the non-myristoylated Z-G2A mutant showed a much more diffused distribution pattern and Z was mainly found to be accumulated in the perinuclear region. (Fig. 4B). Similar results were obtained with Vero cells expressing Z-WT in the presence of the myristoylation inhibitor 2 OHM (Fig. 4C). Our data suggest that punctuate structures targeted by myristoylated Z represent cellular regions important for assembly and budding.

### Inhibition of LASV replication by blocking myristoylation

**Figure 5:**
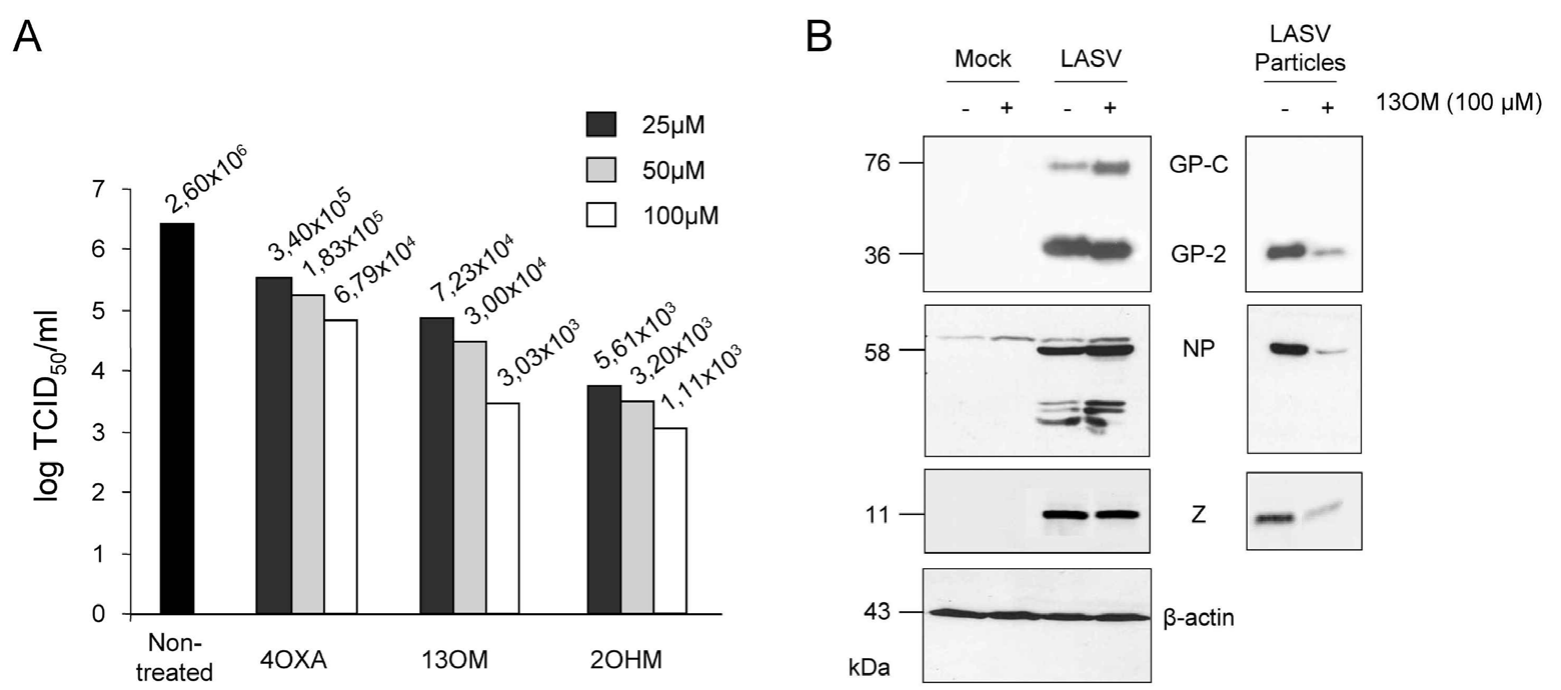
**Effect of myristic acid inhibitors on the release of LASV particles**. Vero cells were infected with LASV (strain Josiah) at a multiplicity of infection of 0.1. At 1 h post-infection, cells were either treated with 3 different myristic acid inhibitors or left untreated as indicated. **A**. 48 hours later cell supernatants were collected and virus yields were determined by TCID_50_. **B**. Virions of cell culture supernatants were passed through a 20% sucrose cushion by ultracentrifugation. Cell lysate and purified virions were subjected to SDS-PAGE followed by immunoblotting using antiserum specific for indicated proteins.

Inhibitors of myristoylation have been reported to inhibit the replication of a wide range of viruses including the Old and New World arenaviruses LCMV and Junin, respectively [17,18,29,31,40-42]. However the impact of blocking myristoylation on LASV replication has not been addressed yet. Therefore we assessed the inhibitory effect of DL-2-hydroxymyristic acid (2 OHM), 13-oxamyristic acid (13 OM) and 4-oxatetradecanoic acid on the multiplication of LASV. Vero cells were infected with LASV and 1 hour post-adsorption, cells were either treated with myristoylation inhibitors at indicated concentrations or left untreated. After 48 hours, supernatants were harvested and virus yields were measured by defining the 50% tissue culture infectious dose (TCID_50_). In the presence of myristoylation inhibitors a significant reduction in virus titers was observed compared to untreated LASV-infected cells indicating that myristoylation plays an important role in the life cycle of LASV (Fig. 5A). DL-2-hydroxymyristic acid and 13-oxamyristic acid exhibited similar effects and reduced virus production up to 3 log_10 _at a final concentration of 100 μM. 4-oxatetradecanoic acid was found to be less active but still able to reduce virus release up to 2 log_10 _compared to untreated infected cells. In order to investigate the effect of blocking myristoylation on virus particle release, cell lysates and supernatants of infected cells were analyzed by SDS-PAGE and immunoblotting. Fig. 5B shows exemplarily the inhibitory potential of 13 OM. The amount of viral proteins in the supernatant of 13 OM-treated cells was highly decreased compared to untreated LASV-infected cells although the intracellular expression levels were similar. Detection of beta-actin was used to demonstrate that treatment of cells with myristic acid inhibitors does not effect cellular protein expression. Similar results were observed for DL-2-hydroxymyristic acid and 4-oxatetradecanoic acid (data not shown). Taken together, our data clearly demonstrate that myristoylation is important for efficient LASV assembly and virus particle release.

## Discussion

In the present report, we analyzed the role of N-myristoylation for the membrane binding properties of LASV Z and its function during virus release. We created a mutant (Z-G2A) that has been shown to fail the incorporation of myristic acid [17] to demonstrate that in the absence of the myristoyl moiety, Z was not efficiently associated with membranes. These results are in agreement with reports for other proteins showing that N-myristoylation is important for membrane binding [36,38,39]. Although the disruption of the myristoylation motif resulted in a clear shift of Z from the membrane to the cytosolic fraction, a minor fraction of Z was still found to be associated with membranes indicating that additional factors might be involved in membrane binding. It is known that many myristoylated proteins like the transforming protein pp60v-src of Rous sarcoma virus or the HIV gag and nef proteins need a cluster of basic amino acids close to the myristoylation site for efficient membrane binding [43-45]. This "myristate plus basic" motif synergizes hydrophobic and electrostatic forces resulting in strong membrane binding. Interestingly, LASV Z contains basic amino acids downstream of the myristoylated glycine residue (Myr-**G**NKQAKAPESKDSPR...) which show some similarity to those present in the N-terminus of the transforming protein pp60v-src (Myr-**G**SSKSKPKDPSQR...) of Rous sarcoma virus. The role of basic amino acids and/or additional hydrophobic sequences within Z for membrane binding is currently under investigation. However, the glycine to alanine exchange was sufficient enough to significantly reduce the release of Z-mediated VLPs. These results are in line with data published by Perez et al. [17] showing that mutation of the glycine at position 2 is critical for VLP formation. Our data suggest that assembly and budding of Lassa virus requires membrane binding of Z to which the myristoyl moiety greatly contributes.

N-terminal myristoylation of LASV Z is not only important for efficient membrane binding but also participate in targeting Z to specific cellular regions. Unlike wild-type Z, the non-myristoylated mutant Z-G2A shows a diffuse distribution pattern throughout the cytoplasm and accumulates in the perinuclear region. The change from a punctuate staining for wild-type to a diffuse cellular distribution for a non-myristoylated mutant has also been described for HIV-1 gag [46]. Our results suggest myristoylation-mediated targeting of Z to specific cellular regions that are important for efficient LASV budding.

The data presented in this work as well as results described by Perez et al. [17] demonstrate that myristoylation of Z plays a key role in arenavirus budding. Treatment of LASV with myristoylation inhibitors resulted in drastic reduction of infectious virus particle production. Although we have shown that myristoylation is crucial for the correct function of Z, it cannot be excluded that other LASV proteins might require myristoylation for their function during virus replication. For the glycoprotein GP of the New world arenavirus Junin virus it was recently shown that its signal peptide is myristoylated and mutation of this myristoylation motif resulted in reduced membrane fusion activity of GP [47]. Similar results were observed for LASV GP (T. Strecker, unpublished results). Thus, myristoylation inhibitors might target multiple arenaviral proteins and therefore may have the potential to serve as an antiviral drug against Lassa virus and other arenaviruses causing hemorrhagic fever in humans.

## Conclusion

Our findings indicate that N-myristoylation of LASV Z plays an important role in both targeting to and association with specific cellular membranes that are important for assembly and budding.

## Materials and methods

### Cell cultures and viruses

Human hepatoma (Huh7) cells and green monkey kidney (VeroE6) cells were cultured in Dulbecco's modified Eagle's medium (DMEM, Gibco) supplemented with 10% fetal calf serum, 100 U/ml penicillin, and 0.1 mg/ml streptomycin. Lassa virus, strain Josiah, was grown on VeroE6 and Huh7 cells, respectively. Infection with Lassa virus was carried out at a MOI of 0.1. All experiments performed with Lassa virus were done under biosafety level 4 biocontainment conditions at the Institute of Virology in Marburg, Germany.

### Molecular cloning and vectorial expression

The open reading frame of Lassa virus Z protein (Lassa virus strain Josiah) was expressed using the pCAGGS vector as described previously [15]. The Z myristoylation mutant, designated Z-G2A, in which the glycine at position 2 was exchanged to alanine was generated by standard PCR techniques. A list of the respective oligonucleotides will be made available on request. The accuracy of all constructs was confirmed by DNA sequencing. Huh7 and VeroE6 cells were transfected with wild type and mutated recombinant DNA using Lipofectamine 2000 (Gibco/Invitrogen).

### Antibodies and reagents

Antisera against GP-C, NP and Z protein were raised by immunization of rabbits as described previously [5,48]. Anti-beta actin antibody was purchased from Abcam (UK). Secondary antibodies conjugated with horse radish peroxidase were purchased from DAKO (USA). Secondary antibodies labelled with Alexa680 (700 nm) were from Molecular Probes™ Invitrogen (Germany) and were used for visualization of proteins using Odyssey™ Infrared Imaging System (Li-Cor Biosciences). Myristic acid inhibitors DL-2-hydroxymyristic acid (2 OHM), 13-oxamyristic acid (13 OM) and 4-oxatetradecanoic acid (4 OXA) were purchased from Sigma-Aldrich (Germany). Stock solutions were prepared in ethanol at 10 mM.

### Acrylamide gel electrophoresis and immunoblotting

Proteins were separated by SDS-PAGE using 12% or 15% polyacrylamide gels as described previously [15]. Immunoblotting was performed as described earlier [49].

### Pulse-chase experiments and immunoprecipitation

Plasmid-transfected Huh7 cells were starved 24 h post-transfection for 1 h with DMEM lacking methionine and cysteine, before cells were labeled with 100 μCi/ml [35S] methionine and [^35^S]cysteine (Amersham). After a 30 min pulse, radioactive medium was replaced by fresh DMEM and cells were chased for different time intervals as indicated. Cells were lysed in Co-IP buffer (1% NP-40, 0.4% deoxycholate (DOC), 5 mM EDTA, 100 mM NaCl, 20 mM Tris-HCl, pH 7.6, 25 mM iodacetamide, 1 mM PMSF). Nuclei and insoluble debris were removed by centrifugation at 14 000 rpm and 4°C. Immunoprecipitation of proteins was performed using protein A-Sepharose-coupled rabbit anti-Z antibodies. Precipitated immunocomplexes were subsequently analyzed by SDS-PAGE followed by autoradiography on BioMax films (Kodak).

### Flotation experiments

Flotation experiments were carried out as described previously [15]. Briefly, Z-expressing Huh7 cells were harvested and disrupted in a hypotonic Tris buffer (20 mM Tris-HCl [pH 7.4]) by 20 strokes of a Dounce homogenizer on ice. Nuclei and cell debris were removed from the cell lysate by low centrifugation at 4°C. OptiPrep (Sigma) was added to the postnuclear supernatant to a final concentration of 35% in a total volume of 500 μl, which was placed at the bottom of a Beckmann-SW60 centrifuge tube. It was overlaid with 3.5 ml of 30% OptiPrep and then with 200 μl of TNE buffer (25 mM Tris-HCl [pH 7.5], 150 mM NaCl, 5 mM EDTA). All OptiPrep solutions were prepared in TNE containing the protease inhibitor mixture Complete (Roche). The gradient was centrifuged to equilibrium at 52 000 rpm for 4 h at 4°C. Fractions were collected from the top, subjected SDS-PAGE and visualized by immunoblotting.

### Generation of Z-induced virus-like particles (VLP)

To generate VLPs, Vero cells were transfected with Lassa virus Z Protein. After 48 h, supernatants cleared from cell debris were laid on a 20% sucrose cushion and ultracentrifuged in a SW-60 rotor at 52 000 rpm at 4°C for 2 h. The pellet was then resuspended in PBS (phosphate-buffered saline) buffer and analyzed by immunoblotting.

### Immunocytochemistry

Vero cells were grown on coverslips and Lipofectamine-transfected with appropriate plasmid constructs for protein expression. 24 hours after transfection, cells were washed and incubated for 5 min with acetone/methanol (1:1 v/v) for fixation and total permeabilization of membranes. Cells were subsequently washed and incubated for 1 h with 1:100 diluted primary rabbit antibodies followed by incubation for 45 min with 1:100 diluted anti-rabbit antibody from goat coupled to FITC (Dianova, Germany). Protein expression of cells was examined using an immunofluorescence microscope (Axiophot, Zeiss, Germany).

### Treatment of LASV-infected cells with myristoylation inhibitors

VeroE6 cells were grown to 80% confluence and infected with LASV at a MOI of 0.1 PFU/cell. 1 hour post-adsorption, cells were either treated with different concentrations of DL-2-hydroxymyristic acid (2 OHM), 13-oxamyristic acid (13 OM) or 4-oxatetradecanoic acid (4 OXA) or left untreated. After 48 hours, cells and supernatants were collected. Virus titration was performed by defining the 50% tissue culture infectious dose (TCID_50_). For this, the supernatants were diluted 5-fold and the dilutions were used to infect VeroE6 cells in 96-well plates (four wells for each dilution). The cultures were scored periodically for cytopathic effect over a period of 7 days. The endpoint virus titers for culture supernatants were calculated with the method of Reed and Muench. Viral titers were expressed as the log_10 _of the 50% titration endpoint for infectivity as calculated by the methods of Karber and Spearman. In addition, the amount of viral proteins released into the supernatant in the presence or absence of myristic acid inhibitors was analyzed by immunoblotting. Therefore, cell lysates and supernatants that were ultracentrifuged as described above were collected and lysed in SDS-PAGE sample buffer.

## Abbreviations

2OHM, DL-2-hydroxymyristic acid; 13OM, 13-oxamyristic acid; 4OXA, 4-oxatetradecanoic acid; GP, glycoprotein; LASV, Lassa virus; MOI, multiplicity of infection; NMT, N-myristoyl transferase; TCID50, tissue culture infecting dose; UC, ultracentrifugation; VLP, virus-like particle.

## Competing interests

The author(s) declare that they have no competing interests.

## Authors' contributions

TS designed the study, carried out experiments, participated in the analysis of the results and wrote the manuscript. AM carried out experiments and participated in the analysis of the results. SD helped to carry out experiments in BSL-4 facility. RE and OL helped to draft the manuscript and revised it critically. WG designed the study, participated in the analysis of the results and helped to draft the manuscript. All authors read and approved the final manuscript.
